# Cardiac repolarization during fingolimod treatment in patients with relapsing–remitting multiple sclerosis

**DOI:** 10.1002/brb3.925

**Published:** 2018-01-30

**Authors:** Aapo Laiho, Tiina M. Laitinen, Päivi Hartikainen, Juha E. K. Hartikainen, Tomi P. Laitinen, Sakari Simula

**Affiliations:** ^1^ Department of Clinical Physiology and Nuclear Medicine Kuopio University Hospital University of Eastern Finland Kuopio Finland; ^2^ Neuro Center Department of Neurology Kuopio University Hospital University of Eastern Finland Kuopio Finland; ^3^ Heart Center Kuopio University Hospital University of Eastern Finland Kuopio Finland; ^4^ Department of Neurology Mikkeli Central Hospital Mikkeli Finland

**Keywords:** cardiac repolarization, Fingolimod, multiple sclerosis, QT‐interval

## Abstract

**Background:**

Fingolimod is a sphingosine‐1‐phosphate receptor modulator for the treatment of relapsing–remitting multiple sclerosis (RRMS). Despite an established effect on heart rate, the effect of fingolimod on cardiac repolarization is not completely known.

**Methods:**

Twenty‐seven patients with RRMS underwent 24‐hr ambulatory ECG before fingolimod (baseline), at the day of fingolimod initiation (1D) and after three‐month treatment (3M). The mean values of RR‐interval as well as QT‐interval corrected by Bazzet's (QTcBaz) and Fridericia's (QTcFri) formula were compared between baseline, 1D, and 3M over 24‐hr period as well as at daytime and nighttime.

**Results:**

QTcBaz over 24‐hr was shorter at 1D (414 ± 20 ms, *p* < .001) and at 3M (414 ± 20 ms, *p* < .001) than at baseline (418 ± 20 ms). In contrast, QTcFri over 24‐hr was longer at 1D (410 ± 19 ms, *p* < .001) but similar at 3M (406 ± 19 ms, *p* = .355) compared to baseline (407 ± 19 ms). Daytime QTcBaz was shorter at 1D (*p* < .001) and at 3M (*p* = .007), whereas daytime QTcFri was longer at 1D (*p* < .05) but similar at 3M (*p* = ns) compared to baseline. During the night, changes were observed neither in QTcBaz nor in QTcFri between baseline, 1D, and 3M.

**Conclusions:**

Changes in cardiac repolarization after fingolimod initiation were mild and occurred at daytime. Ambiguously, QTcBaz demonstrated shortening, whereas QTcFri showed prolongation in cardiac repolarization after fingolimod initiation. The formula applied for QT‐interval correction needs to be taken carefully into account as evaluating pharmacovigilance issues related to fingolimod.

**Highlights**

- Fingolimod is a drug for the treatment of relapsing– remitting multiple sclerosis.
- Effect of fingolimod on cardiac repolarization has not been fully established.
- Fingolimod was found to alter cardiac repolarization predominantly at daytime.
- Analyses based on Bazzet's formula and Fridericia's formula yielded distinct results.
- The results enhance the understanding of drug safety issues related to fingolimod.

## 1 INTRODUCTION

Fingolimod is an oral treatment for relapsing–remitting multiple sclerosis (RRMS). The therapeutic effects of fingolimod on RRMS relate to the modulation of the sphingosine‐1‐phosphate (S1P) receptors (Cohen et al., 2010; Kappos et al., 2010). S1P‐receptor signaling has a role in lymphoid trafficking and also in cardiovascular regulation (Camm, Hla, Bakshi, & Brinkmann, 2014).

The initial cardiac effects of fingolimod resemble those of parasympathetic activation due to S1P1‐receptor agonism (Brinkmann, 2007). Continuous fingolimod dosing, on the other hand, causes down‐regulation of S1P1‐receptors, subsequent shift in S1P‐receptor profile and rebalancing of cardiac autonomic homeostasis (Camm et al., 2014; Simula et al., 2016).

One cardiac cycle consists of depolarization followed by repolarization, which is an active process reestablishing polarity with positive charges on the outer and negative charges on the inner cellular surface (Trenor, Cardona, Saiz, Noble, & Giles, 2017). Myocardial ventricular repolarization is controlled by autonomic nervous system and is reflected by QT‐interval in an electrocardiogram (ECG) (Taggart, Critchley, & Lambiase, 2011). The duration of QT‐interval depends on heart rate and thus needs to be corrected accordingly for adequate comparison. However, results acquired by different correction methods may not be comparable in terms of either mathematical or physiological properties. The value of widely used Bazzet's formula for heart rate correction, for example, has been repeatedly questioned and suggested to be replaced by the Fridericia's formula (U.S. FDA). Prolonged heart rate‐corrected QT‐interval has been demonstrated to be a risk factor for all‐cause mortality, cardiac mortality, and sudden cardiac death (Schouten et al., 1991; Zareba, 2007). On the other hand, short QT‐interval has also been suggested to carry an arrhythmogenic potential (Algra, Tijssen, Roelandt, Pool, & Lubsen, 1993; Viskin et al., 2004).

Although fingolimod has been reported not to prolong heart rate‐corrected QT‐interval significantly (Camm et al., 2014; Rossi et al., 2015; Schmouder et al., 2006), the effects of fingolimod on QT‐interval are not completely established in real life. In this prospective study, we investigated the effects of fingolimod initiation and 3 months of continuous fingolimod treatment on heart rate‐corrected QT‐interval by Bazzet's and Fridericia's formula in real‐life patients with RRMS.

## 2 METHODS

The patients underwent 24‐hr ambulatory ECG recording 20 ± 16 days before fingolimod treatment (baseline), at the day of fingolimod initiation (1D) and after 3 months of (88 ± 7 days) continuous fingolimod treatment (3M). The neurological disability related to RRMS was assessed by Expanded Disability Status Scale (EDSS) for each patient at baseline.

Before participating the study, each patient gave written informed consent after full explanation of the purpose, nature, and risks of all procedures used. The ethics committee of Kuopio University Hospital approved the study protocol. The study was registered at ClinicalTrials.gov (NCT01704183).

### 2.1 Patients

The study consisted of 27 patients with RRMS including 16 (59%) women and 11 (41%) men. Fingolimod was initiated on clinical basis according to the accepted drug label. The first dose of fingolimod was given at hospital before 10:00 a.m. Patients were followed before discharge at least six hours or until heart rate reached the nadir and started to recover. None of the patients needed overnight observation. Initiation of fingolimod was the only change in the medication during the study. All patients had fingolimod as a second‐line treatment for RRMS due to side effects or lack of efficacy during first‐line treatment. Preceding disease‐modifying treatment for RRMS was discontinued at least a day before fingolimod initiation if changed from interferon‐1b or glatirameracetate or at least 2 months before shifting from natalizumab.

The patients were 43 ± 11 years of age, the diagnosis of RRMS was set 10 ± 7 years before the study, and EDSS was 3.4 ± 1.8 on the average. Five patients (19%) had one or more of the following comorbidities: two patients (7%) had type‐1 diabetes mellitus with insulin treatment, three patients (11%) were adequately treated with hormonal substitution for hypothyreosis, one patient (4%) had asthma, and one patient (4%) had optimally treated hypertension.

### 2.2 Acquisition of ECG signal

Twenty‐four‐hour electrocardiogram (ECG) was acquired by ambulatory Schiller Medilog AR12plus recorders (Schiller Medilog, Schiller AG, Switzerland) with a sampling frequency of 250 Hz. Three bipolar ECG leads (modified chest leads V1 and V5 and modified aVF) were used. Digital ECG recordings were read to Darwin Holter analysis system (Schiller Medilog, Schiller AG, Switzerland) and they were exported in MIT‐format for the analyses. Normal daily living was allowed during ambulatory ECG recordings including the six hours in‐hospital observation at the day of fingolimod initiation.

### 2.3 Analysis of ECG

Stationary data sets of 120 s from every hour period, free of technical artifacts and ectopic beats, were selected for analyses. Occasional technical artifacts may confound especially the delicate QT‐interval analysis in ambulatory ECG recordings and thus, the high‐quality 120 s period, well representing the hourly average in the tachogram, was used instead of hourly average. RR‐intervals were analyzed by detecting the time interval between R peaks in adjacent cardiac beats. The QT‐intervals were determined as time elapsed from the onset of Q wave to the end of the T wave. The heart rate‐corrected QT‐interval was computed according to the Bazett's formula $\text{QTcBaz}=\text{QT}/\sqrt{\text{RR}-\text{interval}}$ and by the Fridericia's formula $\text{QT cFri}=\text{QT}/\sqrt[{3}]{\text{RR}-\text{interval}}$. Data analyses were performed with WINCPRS software (Absolute Aliens Ltd, Turku, Finland) (Kuusela, Jartti, Tahvanainen, & Kaila, 2005), and reliability of automated data analyses was controlled by visual evaluation of the ECG signal.

### 2.4 Statistical analyses

The normal distribution of values was assessed by Kolmogorov–Smirnov test. Repeated measures linear mixed model was applied to study differences and significances between specific time points at baseline, 1D, and 3M. Results were analyzed separately on 24‐hr period, daytime (first 12 hours of the recording) and nighttime (12 p.m.–8 a.m.). Results were expressed as mean ± standard deviation (*SD*) unless otherwise indicated. All analyses were conducted at the two‐tailed level and a *p‐*value <.05 was considered statistically significant. All statistical analyses were performed using IBM SPSS statistics (version 21, 1989‐2012 SPSS Inc, Chicago, USA).

## 3 RESULTS

### 3.1 Changes in RR‐interval

**Figure 1:**
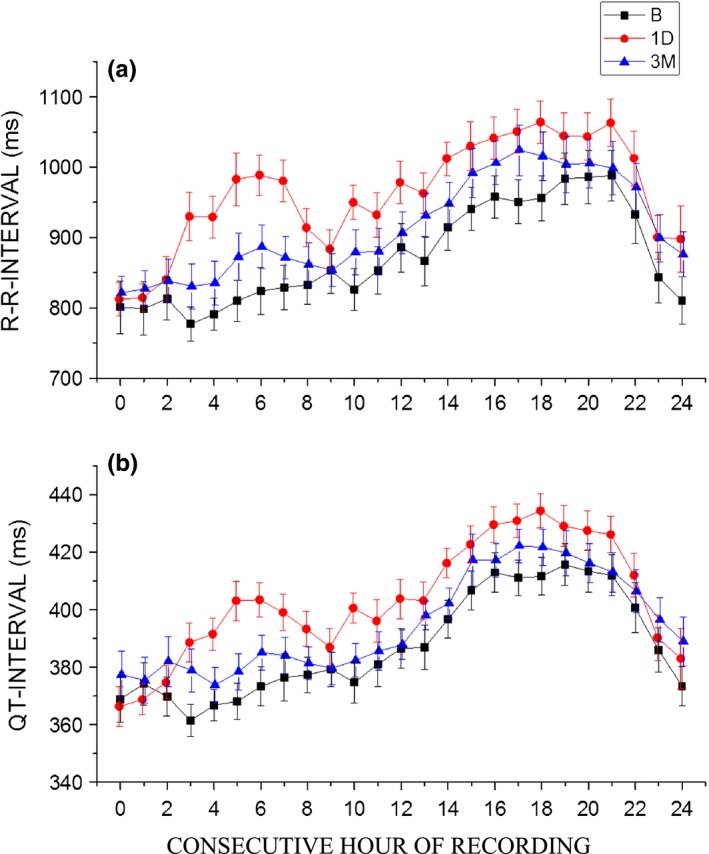
The 24‐hr trend in RR‐interval (panel a) and QT‐interval (panel b) before fingolimod initiation at baseline (B), at the day of fingolimod initiation (1D) and after three months of treatment (3M) (*n *= 27). The values are mean ± *SEM*

The 24‐hr RR‐interval was longer (*p* < .001) at 1D than at baseline (Table 1, Figure 1a). At 3M, the 24‐hr RR‐interval was shorter (*p* < .001) than at 1D, but still longer (*p* < .001) than at baseline (Table 1, Figure 1a). The prolongation of the 24‐hr RR‐interval from baseline was 88.7 ± 32.1 ms (10.2 ± 3.7%) to 1D and 36.7 ± 33.4 ms (4.2 ± 3.8%) to 3M.

**Table 1:** The mean values of electrocardiogram parameters over 24‐hr period (*n *= 27)

	B	1D	3M	*p*‐value B vs 1D	*p*‐value B vs 3M	*p*‐value 1D vs 3M
RRI (ms)	872 ± 120	961 ± 120	909 ± 120	<.001	<.001	<.001
QT (ms)	387 ± 26	403 ± 26	392 ± 26	<.001	<.001	<.001
QTcBaz (ms)	418 ± 20	414 ± 20	414 ± 20	<.001	<.001	.662
QTcFri (ms)	407 ± 19	410 ± 19	406 ± 19	<.001	.355	<.001

B, baseline; QT, QT‐interval; QTcBaz, QTc interval corrected by Bazett's formula; QTcFri, QTc interval corrected by Fridericia's formula; RRI, interval between two consecutive R peaks; 1D, the day of fingolimod initiation; 3M, three months. Values are mean ± *SD*.

During the day, RR‐interval was 816 ± 116 ms at baseline and was found longer at 1D (911 ± 116 ms; *p* < .001). At 3M, daytime RR‐interval was shorter (850 ± 117 ms) as compared to 1D (*p* < .001) but still longer than at baseline (*p* < .001).

During the night, RR‐interval was 957 ± 144 ms at baseline and was found longer at 1D (1035 ± 144ms; *p* < .001). At 3M, nighttime RR‐interval was shorter (991 ± 145 ms; *p* < .001) as compared to 1D but still significantly longer than at baseline (*p* = .001).

### 3.2 Changes in QT‐interval

The 24‐hr QT‐interval was longer at 1D (*p* < .001) than at baseline (Table 1, Figure 1b). At 3M, the 24‐hr QT‐interval was shorter (*p* < .001) as compared to 1D but still longer than at baseline (*p* < .001) (Table 1, Figure 1b). The prolongation in 24‐hr QT‐interval from baseline was 16.0 ± 6.7 ms (4.1 ± 1.7%) to 1D and 4.9 ± 7.0 ms (1.3 ± 1.8%) to 3M.

During the day, QT‐interval was 372 ± 26 ms at baseline and was found longer at 1D (389 ± 27 ms; *p* < .001). At 3M, daytime QT‐interval was shorter (377 ± 27 ms; *p* < .001) as compared to 1D but still significantly longer as compared to baseline (*p* < .01). The prolongation in daytime QT‐interval from baseline was 17.0 ± 9.2 ms (4.6 ± 2.5%) to 1D and 5.3 ± 9.6 ms (1.4 ± 2.6%) to 3M.

During the night, QT‐interval was 410 ± 29 ms at baseline and was found longer at 1D (424 ± 29 ms, *p* < .001). At 3M, nighttime QT‐interval was shorter (414 ± 29 ms; *p* < .001) than at 1D but showed no difference to baseline (*p* = ns). The prolongation in nighttime QT‐interval from baseline was 14.2 ± 10.4 ms (3.5 ± 2.5%) to 1D.

### 3.3 Changes in QT‐interval corrected by Bazett's formula

**Figure 2:**
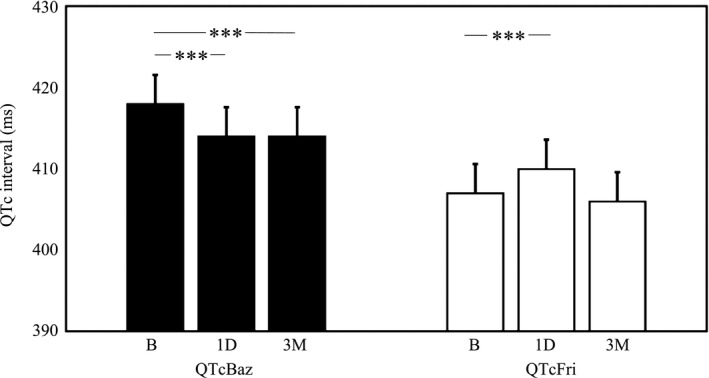
Heart rate‐corrected QT‐interval over 24‐hr period before fingolimod initiation at baseline (B), at the day of fingolimod initiation (1D), and after three months of treatment (3M) as calculated by Bazzet's (QTcBaz) and Fridericia's (QTcFri) formula. The values are mean ± *SEM*. Significance: *** = *p* < .001

The 24‐hr QTcBaz was shorter at 1D (*p* < .001) and at 3M (*p* < .001) as compared to baseline (Table 1, Figure 2). From baseline, the 24‐hr QTcBaz shortened 3.9 ± 4.7 ms (0.9 ± 1.2%) to 1D and 4.3 ± 4.9 ms (1.0 ± 1.2%) to 3M. The 24‐hr QTcBaz did not show difference between 1D and 3M (*p* = ns).

During the day, QTcBaz was found shorter at 1D (411 ± 21 ms; *p* < .001) as well as at 3M (411 ± 21 ms; *p* = .007) than at baseline (415 ± 21 ms). From baseline, daytime QTcBaz shortened 4.6 ± 6.9 ms (1.1 ± 1.7%) to 1D and 3.8 ± 7.2 ms (0.9 ± 1.8%) to 3M, on the average. There was no difference in daytime QTcBaz between 1D and 3M (*p* = ns.). During the night, there was no difference (*p* = ns) in QTcBaz between baseline (422 ± 21 ms), 1D (419 ± 21 ms), and 3M (418 ± 21 ms).

### 3.4 Changes in QT‐interval corrected by Fridericia's formula

The 24‐hr QTcFri during 24‐hr was longer at 1D (*p* < .001) than at baseline (Table 1, Figure 2). From baseline, the prolongation in QTcFri was 3.0 ± 4.3 ms (0.7 ± 1.1%) to 1D. At 3M, the 24‐hr QTcFri was 406 ± 19ms and did not differ from that at baseline (*p* = ns).

During the day, QTcFri was 400 ± 20 ms at baseline and was found longer at 1D (403 ± 20 ms; *p* < .05). QTcFri prolonged 3.0 ± 6.0 ms (0.8 ± 1.5%) from baseline to 1D.

During the night, there was no difference (*p* = ns) in QTcFri between baseline (418 ± 20 ms), 1D (421 ± 20 ms), and 3M (416 ± 20 ms).

## 4 DISCUSSION

This study demonstrated that changes in heart rate‐corrected QT‐interval in patients with RRMS after fingolimod initiation are mild and occurred predominantly at daytime. Ambiguously, Bazzet's and Fridericia's formula for heart rate correction of QT‐interval yielded incomparable results.

Fingolimod initiation resulted in shorter QT‐interval in patients with RRMS when heart rate correction was based on Bazett's formula. Shorter heart rate‐corrected QT‐interval was found at the day of fingolimod initiation as well as after 3 months of continuous fingolimod treatment. Previously, to our knowledge, the effect of fingolimod on Bazett‐corrected QT‐interval has not been reported. Accordingly, mild but long‐lasting shortening in heart rate‐corrected QT‐interval was demonstrated during fingolimod treatment for the first time.

Previously, mild prolongation in heart rate‐corrected QT‐interval has been reported in a small proportion of patients with RRMS after fingolimod initiation when calculations were based on Fridericia's formula (Camm et al., 2014; Rossi et al., 2015). Correspondingly, in our study, QT‐interval corrected by Fridericia's formula demonstrated mild prolongation at the day of fingolimod initiation. After 3 months of continuous fingolimod treatment, on the other hand, Fridericia‐corrected QT‐interval did not show any more difference to the values at baseline (before fingolimod initiation) thus suggestive of complete recovery.

Regardless of the applied correction formula, heart rate‐corrected QT‐interval was found to change as a result of fingolimod initiation at daytime. Previously, cardiac responses after fingolimod initiation have been shown to depend on the prevailing cardiac autonomic tone (Rossi et al., 2015). This is well in line with our present finding that the effect of fingolimod on heart rate‐corrected QT‐interval was different at daytime and nighttime. One possible explanation for this novel finding relies on the physiological circadian fluctuation in cardiac autonomic tone, that is, prevailing parasympathetic tone at nighttime, and prevailing sympathetic tone at daytime. In other words, the effect of S1P‐receptor modulation by fingolimod initiation on cardiac repolarization is different during sympathetic predominance (at daytime) and vagal predominance (nighttime).

The initial S1P1‐receptor agonism of fingolimod mimics that of parasympathetic activation (Egom, Kruzliak, Rotrekl, & Lei, 2015). Decrease in heart rate (equal to prolongation in RR‐interval), prolongation in atrioventricular conduction, and increase in parasympathetic components of cardiac autonomic regulation have been demonstrated shortly after the first dose of fingolimod (Cohen et al., 2010; Simula et al., 2015). In the present study, QT‐interval corrected by Bazett's formula was found shorter, whereas Fridericia‐corrected QT‐interval demonstrated prolongation at the day of fingolimod initiation. Thus, the direction of change in heart rate‐corrected QT‐interval depends ambiguously on the applied mathematical correction formula.

Continuous fingolimod treatment results in internalization of S1P1‐receptors and subsequently shifts the S1P‐receptor profile toward S1P2 and S1P3 dominance. Previously, uncoupling between heart rate and corresponding cardiac autonomic tone has been demonstrated during continuous fingolimod therapy (Simula et al., 2016). In the present study, heart rate‐corrected QT‐interval remained slightly shorter (Bazzet's formula) or unchanged (Fridericia's formula) regardless of the lower heart rate (i.e. prolonged RR‐interval) after 3 months of continuous fingolimod treatment. This finding suggests that shift in S1P‐receptor profile during continuous fingolimod treatment has different effect on the regulation of cardiac repolarization and heart rate (depolarization).

The duration of QT‐interval depends on heart rate and needs to be corrected accordingly. Different correction formulae have been introduced, but every method has faced‐specific criticism. For example, widely used Bazett's formula tends to overcorrect QT‐interval at faster and undercorrect at slower heart rates, whereas the opposite bias is considered for Fridericia's formula. Medication altering the physiological basis of heart rate and QT‐interval regulation, such as fingolimod, may challenge the interpretation of changes in cardiac repolarization even more. Previously, mathematical QT/RR relation that fits for all subjects individually has been stated unobtainable (Malik, Färbom, Batchvarov, Hnatkova, & Camm, 2002). However, as resting heart rate usually fluctuates between 60 and 90 bpm, the U.S. Food and Drug Administration has recommended to use Fridericia's formula in clinical trials on drug safety (U.S. Food and Drug Administration). Recently, use of Fridericia's formula instead of Bazett's formula has also been recommended in the prediction of mortality (Vandael, Vandenberk, Vandenberghe, Willems, & Foulon, 2017).

Activity of autonomic nervous system fluctuates according to circadian cycle (Huikuri et al., 1994). It is not possible to standardize this phenomenon even in tightly controlled laboratory environment. In our study, 24‐hr ECG recording was undertaken during normal daily activities. As recording was started during morning hours (at different time on different subjects but before 10:00 a.m. in any case), we judged that first 12 hrs of recording represent daytime (awake‐hours). On the other hand, nighttime (sleep‐hours) was defined according to the clock from midnight to 08 a.m. as every patient had this time period in their recording. We consider that other definitions for daytime and nighttime might have worked as well and would not have had significant effect on the results.

## 5 CONCLUSION

Fingolimod modulates cardiac repolarization in patients with RRMS. Changes in cardiac repolarization appear mild and occur at daytime predominantly. After fingolimod initiation, heart rate‐corrected QT‐interval shows long‐lasting shortening when analysis is based on Bazzet's formula but only transient prolongation when analysis is based on Fridericia's formula. Accordingly, the formula applied for QT‐interval correction needs to be taken into account by all stakeholders as evaluating pharmacovigilance issues related to fingolimod. In addition, these new findings contribute significantly to the understanding of the effects of S1P‐receptor modulation on cardiac repolarization in patients with RRMS.

## CONFLICT OF INTEREST

AL: None. TML: None. PH has been the congress representative of Kuopio University Hospital sponsored by industry (BiogenIdec, Genzyme, TEVA). JEKH has received research grants from the Finnish Foundation for Cardiovascular Research and the European Union Seventh Framework Programme and has been a speaker sponsored by industry (Cardiome, Biotronic, Amgen, MSD, AstraZeneca). TPL has received a research grant from the Finnish Foundation for Cardiovascular Research. SS has been the congress representative of Mikkeli Central Hospital sponsored by industry (BiogenIdec, Boehringer Ingelheim, Genzyme, GlaxoSmithKline, Merck, Novartis, OrionPharma, Sanofi, TEVA), has been a speaker sponsored by industry (BiogenIdec, Genzyme, Merck, Novartis, TEVA) and has received consultancy fees from industry (BiogenIdec and Merck).
